# Construction of a visual nomogram prediction model for post-traumatic stress disorder in mothers of very low birth weight infants

**DOI:** 10.3389/fpsyt.2025.1550267

**Published:** 2025-07-16

**Authors:** Liuyi Lu, Yujiao Xu, Xiaolong Jin

**Affiliations:** Shandong Provincial Hospital Affiliated to Shandong First Medical University, Jinan, China

**Keywords:** very low birth weight infants, mothers, post-traumatic stress disorder, PTSD, nomogram, risk prediction, neonatal intensive care

## Abstract

**Introduction:**

The prevalence of very low birth weight infants (VLBWIs) continues to rise globally. These infants face increased susceptibility to preterm-related complications and potential long-term chronic health conditions, creating significant psychological and economic burdens for their families. Mothers of VLBWIs demonstrate substantially higher risks of developing post-traumatic stress disorder (PTSD) compared to mothers of term infants. Maternal PTSD can impair psychosocial functioning, compromise therapeutic relationships, and disrupt early mother–infant bonding, with potentially lasting consequences for child development. These findings highlight the critical importance of early PTSD screening and intervention in this vulnerable population.

**Methods and analysis:**

We enrolled 352 mothers of very low birth weight infants (<1,500 g) admitted to NICUs at three hospitals in Shandong Province between September 2022 and December 2023. We identified PTSD risk factors through multivariable logistic regression and developed predictive models based on these results. Model validation included ROC analysis, calibration curves, and decision curve analysis.

**Results:**

Among the 352 mothers of preterm infants, the PTSD detection rate was 40.9%. Logistic regression analysis identified six independent risk factors for PTSD in mothers of VLBWIs: history of miscarriage, spousal support, illness uncertainty, neurotic personality traits, negative coping styles, and perceived social support. The nomogram model developed from these factors demonstrated excellent discriminative ability, with an area under the ROC curve of 0.969 (95% CI: 0.953–0.984). Both the Hosmer–Lemeshow goodness-of-fit test (*P* = 0.956) and calibration curves indicated strong agreement between predicted and observed outcomes.

**Discussion:**

Understanding the prevalence of PTSD among mothers of extremely low birth weight infants, identifying its independent risk factors, and developing a visual nomogram prediction model are critical for enabling early PTSD detection and facilitating clinical screening of high-risk mothers.

## Introduction

Very low birth weight infants (VLBWIs), defined as neonates weighing <1,500 g at birth (1), represent a critical population in neonatal intensive care units (NICUs). Accounting for approximately 20% of NICU admissions (2), their prevalence continues to rise. Compared to term infants, VLBWIs demonstrate significantly higher risks of immediate complications, including thermoregulatory instability, hypoglycemia, and respiratory distress (3), often requiring hospital readmission.

Long-term outcomes may include motor and sensory impairments, neurocognitive deficits, and behavioral disorders (4). These complications contribute to elevated morbidity and mortality rates, increased healthcare expenditures, and prolonged hospitalizations. Furthermore, they impose substantial psychological burdens on parents, particularly predisposing mothers to adverse mental health outcomes including post-traumatic stress disorder.

Post-traumatic stress disorder (PTSD) is a severe psychiatric condition that can develop after exposure to a traumatic event. PTSD is often conceptualized as a single diagnosis without further distinction. However, PTSD presentations vary widely and are classified into several subtypes based on symptom onset and duration. These include acute PTSD (symptoms lasting less than 3 months), chronic PTSD (symptoms persisting for 3 months or longer), delayed-onset PTSD (symptoms emerging ≥6 months after the event), and complex PTSD (stemming from prolonged or repeated trauma). PTSD is clinically diagnosed when the PCL-C score exceeds 38 points following exposure to traumatic events, with symptoms manifesting across three core dimensions: re-experiencing, avoidance/numbing, and hyperarousal (5, 6). Research indicates that 23%–35% of NICU mothers of preterm infants develop PTSD (7), with national studies reporting a prevalence of 33.1%.

Mothers of very low birth weight infants experience significantly greater financial stress compared to mothers of term infants (8). International studies demonstrate that maternal PTSD can lead to maladaptive parenting behaviors, including excessive control tendencies (9, 10) and impaired mother–infant attachment (11). Longitudinal studies reveal that children of mothers with perinatal psychological disorders (including PTSD and depression) exhibit 2–5 times higher rates of behavioral difficulties (12), with increased risks of social withdrawal, anxiety, and peer aggression during school years (13).

These findings underscore the critical importance of early PTSD identification in mothers of very low birth weight infants.

## Objectives

1. To investigate the prevalence and identify independent risk factors for PTSD among mothers of ELBWIs
2. To develop and validate a nomogram prediction model for PTSD risk assessment in this population

### Preparation

A multidisciplinary research team was assembled, consisting of two neonatologists/gynecologists, one psychiatrist, three nursing specialists, one biostatistician, and three research assistants. All team members underwent standardized training. Through evidence-based literature review and clinical practice integration, the team developed a comprehensive understanding of PTSD in mothers of preterm infants. We conducted multiple rounds of methodological discussions to ensure both the feasibility of the research approach and proper implementation at each study phase.

### Participants

This multicenter study will be conducted at several grade III, level A tertiary hospitals in Shandong Province. These study sites were selected based on:

1. High-volume neonatal admissions, ensuring an adequate sample size of preterm infants
2. Established track record of excellent family compliance
3. Comprehensive research infrastructure and data resources

### Patient recruitment

This study employed convenience sampling to recruit mothers of ELBWIs (<1,500 g) admitted to NICUs of three tertiary hospitals in Shandong Province (including Shandong Provincial Hospital) between September 2022 and September 2023.

### Eligibility criteria

#### Inclusion criteria

Infant factors: birth weight <1,500 g; gestational age <32 weeks

Maternal factors: willing participation with signed informed consent

#### Exclusion criteria

Infant factors: hospital discharge outcomes: abandonment, transfer, or mortality

Maternal factors: 1) history of other significant traumatic exposures (e.g., witnessing violent death or serious accidents) and 2) pre-existing psychiatric conditions impairing study participation

### Sample size calculation

We determined the minimum required sample size using established logistic regression parameters:

Key parameters:

­ 17 candidate predictor variables identified from the literature
­ Minimum of 5–10 events required per variable (conservatively using 5)
­ Estimated PTSD prevalence range: 26%–53% (using th upper bound 53% for robust estimation)
­ 20% buffer for potential data attrition

Calculation:


$$Minimum\;sample\;size=\left(Variables\times Events\;per\;variable\right)÷\left(Prevalence\times Valid\;response\;rate\right)=\left(17\times 5\right)÷\left(0.53\times 0.8\right)=200\;participants$$

Rationale:

1. The calculation follows Peduzzi et al.’s recommendation for logistic regression studies.
2. Using the upper prevalence bound ensures adequate power across potential incidence rates.
3. The 20% attrition buffer accounts for potential missing data or withdrawals.

Final determination: A minimum of 200 mother–infant dyads were required to ensure sufficient power for detecting significant predictors while maintaining statistical reliability.

### Measurement tool

1) Participants were assessed for PTSD using standardized diagnostic criteria and questionnaires. PTSD diagnosis was based on the DSM-5 criteria, which require A) exposure to actual or threatened death, serious injury, or sexual violence; and B–E) the presence of one or more intrusive re-experiencing symptoms, one or more avoidance symptoms, two or more negative alterations in cognition or mood, and two or more hyperarousal symptoms, persisting for at least 1 month after the trauma. We administered the PTSD Checklist-Civilian version (PCL-C), a 17-item self-report measure of PTSD symptoms. Each item was rated on a 5-point scale (1 = “not at all” to 5 = “extremely”), yielding a total score from 17 to 85. Following published guidelines, we used a cutoff score of 38 to indicate probable PTSD (14).

The neuroticism subscale of the Five-Factor Inventory (FFI-N) was administered to assess maternal personality traits. This validated 12-item instrument uses a 5-point Likert scale, with higher composite scores (range: 12–60) indicating greater neurotic tendencies. The scale demonstrates excellent reliability in our sample (Cronbach’s *α* = 0.89). The 2) Perceived Social Support Scale (PSSS) and the Parents’ Perception of Uncertainty in Illness Scale (PPUS) were also administered.

3) In addition to PTSD measures, we systematically recorded perinatal stressors experienced by each participant. These included obstetric complications, delivery factors, and neonatal issues. The choice of these stressors was guided by prior literature identifying such events as risk factors for postpartum PTSD (15–17). Other relevant measures (e.g., demographic and clinical variables) were collected according to standard protocols.

## Results

### PTSD symptom profiles

Mothers of preterm infants exhibited PTSD symptoms with the following characteristics: total PCL-C scores: from 17 to 74 (mean 38.6 ± 11.26) and symptom cluster scores: 1) re-experiencing: 10.36 ± 4.06, 2) avoidance/numbing: 7.80 ± 2.99, and 3) hyperarousal: 13.60 ± 4.21.

### Group comparisons

Univariate analysis identified significant differences (*P* < 0.05) between PTSD and control groups across four domains:

1. Demographic factors: maternal age, household income
2. Obstetric history: assisted conception, delivery mode, miscarriage history, prior psychiatric diagnosis
3. Neonatal factors: infant birth weight, NICU length of stay
4. Psychosocial measures: illness uncertainty, neuroticism, coping strategies (positive/negative), perceived social supp

The complete statistical results are presented in **Table 1**.

**Table 1:** Results of one-way analysis of PTSD.

Sports event	Total sample (*n* = 352)	PTSD group (*n* = 144)	Non-PTSD group (*n* = 208)	Statistical test	*P*-value
(A person’s) age	32 (28, 35)	33 (29, 36)	31 (28, 34)	−2.789	<0.01
Monthly household income				14.468	<0.01
4,000 and below	37 (10.51%)	16 (4.55%)	21 (5.97%)		
4,000–6,000	128 (36.36%)	65 (18.47%)	63 (17.90%)		
6,000–8,000	99 (28.13%)	41 (11.65%)	58 (16.48%)		
8,000 and above	88 (25%)	22 (6.25%)	66 (18.75%)		
Educational attainment				5.455	0.065
High school and below	131 (37.22%)	64 (18.18%)	67 (19.03%)		
College and bachelor’s degree	213 (60.51%)	77 (21.88%)	136 (38.64%)		
Postgraduate and above	8 (2.27)	3 (0.85%)	5 (1.42%)		
Type of pregnancy				9.735	<0.01
Natural conception	278 (79%)	102 (28.98%)	176 (50%)		
*In-vitro* fertilization	74 (21%)	42 (11.93%)	32 (9.09%)		
Previous psychiatric history				5.973	<0.01
Present	12 (3.41%)	9 (2.56%)	3 (0.85%)		
Absent	340 (96.59%)	135 (38.35%)	205 (58.24%)		
Complications during pregnancy				0.896	0.344
Present	180 (51.14%)	78 (22.16%)	102 (28.98%)		
Absent	172 (48.86%)	66 (18.75%)	106 (30.11%)		
Mode of delivery				4.942	0.026
Natural childbirth	26 (7.39%)	16 (4.55%)	10 (2.84%)		
Cesarean section	326 (92.61%)	128 (36.36%)	198 (56.25%)		
Planned pregnancy				1.411	0.235
Present	257 (73.01%)	110 (31.25%)	147 (41.76%)		
Absent	95 (26.99%)	34 (9.66%)	61 (27.33%)		
Complications of prematurity				0.896	0.344
Present	180 (51.14%)	78 (22.16%)	102 (28.98%)		
Absent	172 (48.86%)	66 (18.75%)	106 (30.11%)		
Number of abortions	1 (0, 1)	1 (0, 2)	1 (0, 1)	−4.945	<0.01
Birth weight of premature babies	1,295 (1,000, 1,450)	1,085 (900, 1,340)	1,390 (1,122.5, 1,450)	−7.741	<0.01
Length of hospitalization	38 (28, 57)	45 (30, 63)	34 (25, 47)	−4.003	<0.01
Disease uncertainty	85 (76, 98)	98 (89, 115)	77 (47, 85)	−13.119	<0.01
Uncertainty	29 (25, 37)	37 (29, 49)	27 (24, 32)	−9.493	<0.01
Sophistication	24 (21, 28)	28.7 ± 5.766	21 (20, 24)	−10.555	<0.01
Lack of information	17 (15, 19)	19 (16, 20)	16 (15, 18)	−4.938	<0.01
Unpredictability	14 (11, 15)	15 (14, 17)	12 (10, 14)	−10.777	<0.01
Neuroticism	33 (29, 36)	35 (33, 39)	31 (27, 34)	−8.419	<0.01
Positive ways of responding	31 (28, 34)	30 (27, 33)	32 (29, 34)	−2.575	<0.01
Negative coping styles	19 (16, 21)	20 (18, 22)	17 (14, 17)	−6.564	<0.01
Social security (pensions, medical insurance)	60 (51, 70)	52 (46, 60)	68.5 (57, 73)	−9.714	<0.01

### Collinearity diagnostics

We assessed multicollinearity among predictor variables using two metrics: 1) tolerance values (>0.1) and 2) variance inflation factors (VIFs < 5). Diagnostic results confirmed the absence of multicollinearity concerns across all 13 examined variables, supporting their independent inclusion in subsequent regression analyses.

### Multicollinearity assessment

We evaluated potential multicollinearity among predictors using tolerance values and VIFs. All 13 candidate variables demonstrated acceptable ranges (tolerance > 0.1; VIF < 5), indicating no substantial multicollinearity concerns.

### Logistic regression analysis

We constructed a binary logistic regression model with maternal PTSD status as the outcome variable. All 13 variables showing significant associations (*P* < 0.05) in the univariate analyses were entered as predictors after appropriate coding (see **Table 2**). The final regression model results are presented in **Table 3**, identifying significant independent risk factors for PTSD development in mothers of preterm infants.

**Table 2:** Logistic regression assignment table.

Variable name	Variable assignment
X1 Previous family history of mental illness	No = 0; yes = 1
X2 Pregnancy methods	Natural conception = 1; *in-vitro* fertilization = 2
X3 Mode of delivery	Normal birth = 1; cesarean section = 2
X4 Monthly household income	Below $4,000 = X4 (1)
	4,000–6,000 = X4 (2)
	6,000–8,000 = X4 (3)
	8,000 and above = X4 (4)
X5 Age	Original value entry
X6 Number of abortions	Original value entry
X7 Birth weight	Original value entry
X8 Length of stay	Original value entry
X9 Disease uncertainty	Original value entry
X10 Neurotic personality	Original value entry
X11 Positive response approach	Original value entry
X12 Negative coping styles	Original value entry
X13 Support score	Original value entry
Y Post-traumatic stress disorder	No = 0; yes = 1

**Table 3:** Binary logistic regression analysis for PTSD.

Predictor variable	*B*	SE	Wald	*P*	OR	95% CI
Number of abortions	0.816	0.243	11.261	0.001	2.262	1.404–3.644
Birth weight	−0.004	0.001	16.769	0	0.996	0.995–0.998
Disease uncertainty	0.136	0.02	45.789	0	1.145	1.101–1.191
Negative coping styles	0.265	0.073	13.118	0	1.304	1.129–1.505
Neuroticism	0.157	0.042	13.807	0	1.171	1.077–1.272
Total support score	−0.087	0.021	16.653	0	0.917	0.879–0.956
Constant	−13.934	3.141	19.673	0	0	

### Predictive nomogram development

**Figure 1:**
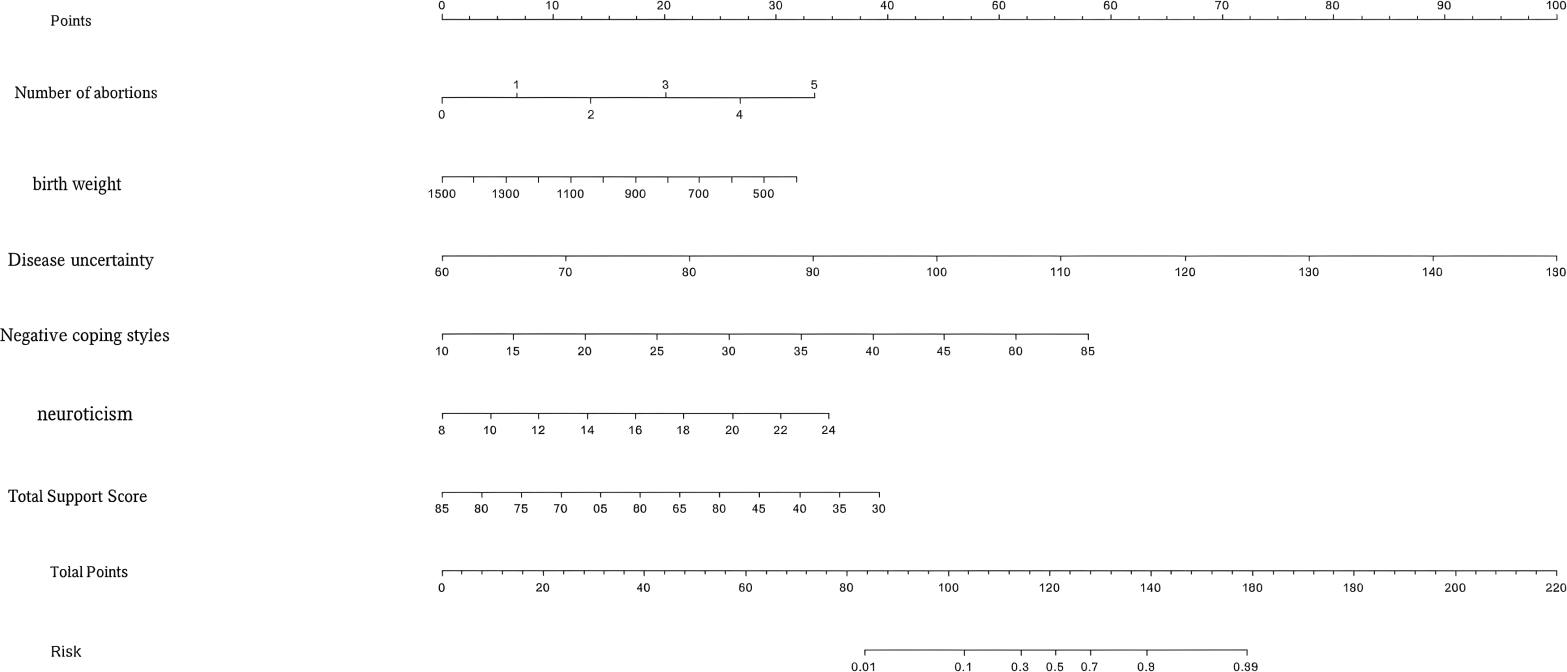
Individualized nomogram early warning model for PTSD in very low birth weight infants.

We developed a clinically applicable nomogram (**Figure 1**) to assess PTSD risk in mothers of VLBWIs, incorporating all significant predictors identified through our multivariable logistic regression analysis. This visual predictive tool enables individualized risk estimation, immediate clinical assessment, and stratification of high-risk patients.

### Model discrimination performance

**Figure 2:**
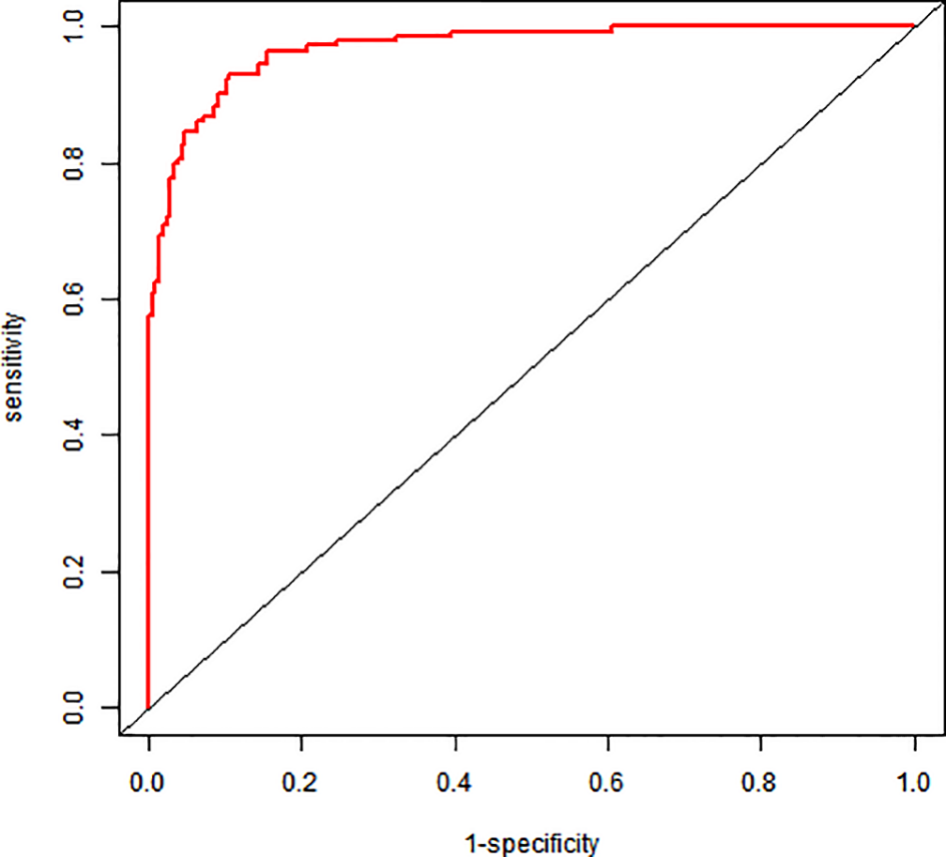
ROC graph for a column-line graph.

The nomogram demonstrated excellent discriminative ability: AUC = 0.969 (95% CI: 0.953–0.984); sensitivity: 93.1%; specificity: 89.4%; optimal cutoff: 0.356 (Youden index = 0.825) (**Figure 2**).

### Model validation

**Figure 3:**
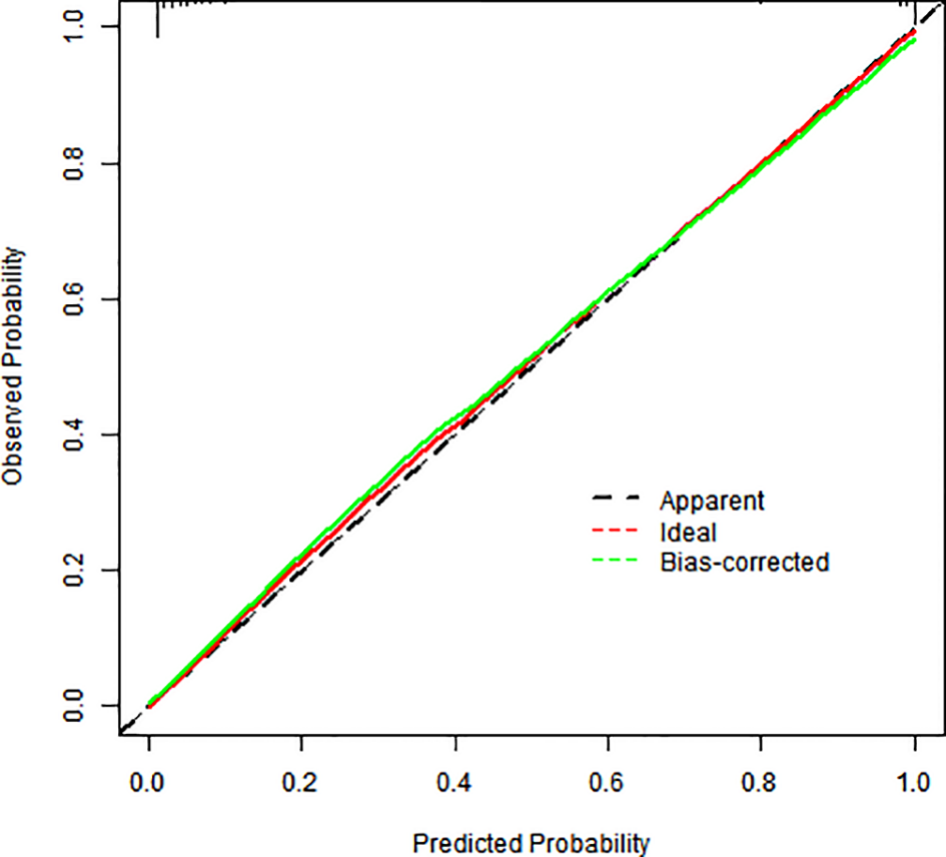
Calibration graph for a column-line graph.

Internal validation via bootstrap resampling (1,000 iterations) confirmed model robustness: Hosmer–Lemeshow test: *χ*² = 2.6128, *P* = 0.956. The calibration curves showed excellent agreement between predicted and observed risks (**Figure 3**).

### Clinical applicability

**Figure 4:**
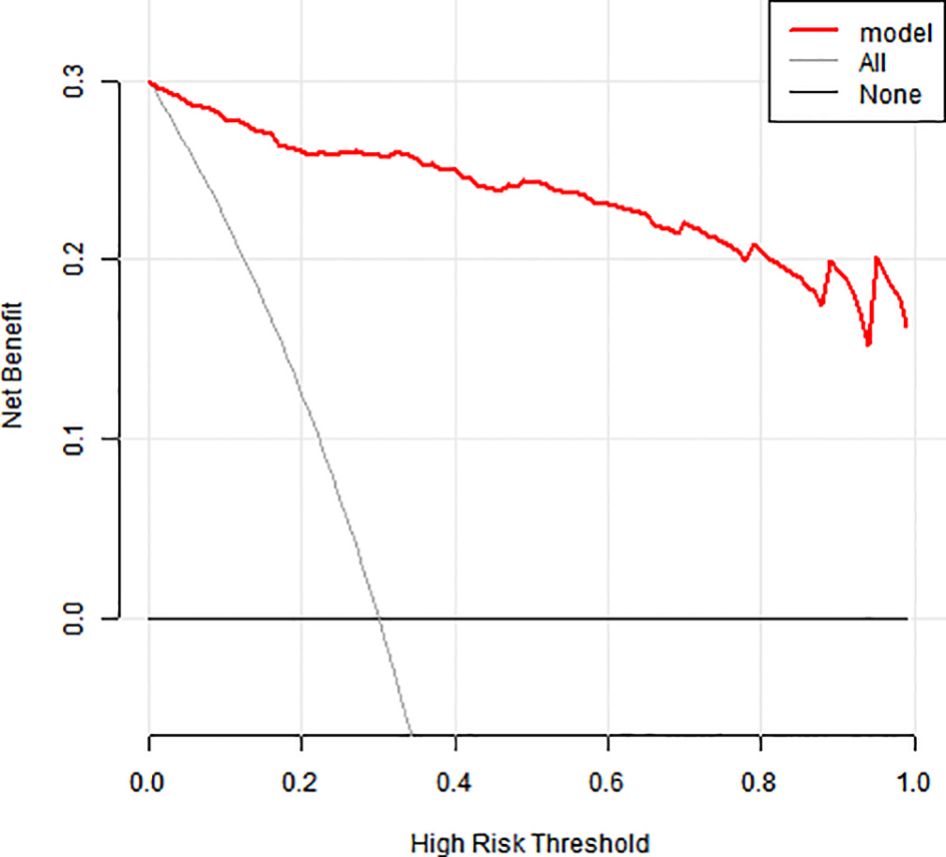
Clinical decision-making curve for a column-line diagram.

**Figure 5:**
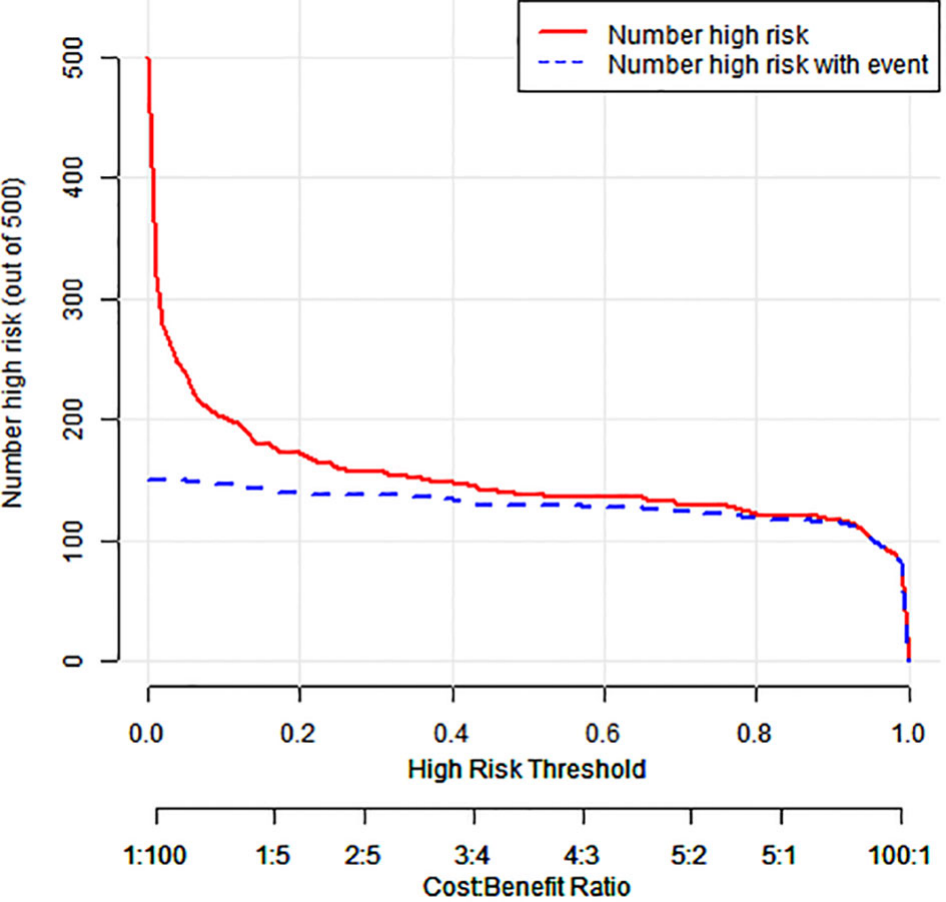
Clinical impact curve for a column-line diagram.

The model showed 1) strong predictive accuracy for PTSD risk, 2) excellent clinical utility per decision curve analysis (**Figure 4**), and 3) significant clinical value across multiple thresholds (**Figure 5**).

## Discussion

Our findings indicate that neurotic personality traits represent a significant risk factor for PTSD among mothers of preterm infants, with higher neuroticism scores correlating with increased PTSD susceptibility. This aligns with the findings of Mattson (18), showing that neuroticism serves as both a psychological vulnerability factor for stress-related disorders and one of the strongest predictors of PTSD, characterized by negative emotionality and affective instability. Individuals with high neuroticism exhibit heightened emotional reactivity to stressors, demonstrating greater negative affect volatility and predisposition to anxiety/impulsivity compared to the general population. Consequently, early targeted psychological interventions by healthcare providers could enhance stress resilience in these mothers, potentially mitigating PTSD incidence through improved coping capacity and emotional regulation.

Existing literature (19) demonstrates that prior pregnancy loss (including spontaneous abortion, stillbirth, and/or induced abortion) significantly increases preterm mothers’ risk of postpartum depression, anxiety, and PTSD. This finding aligns with the study of Aftyka (20), showing significant positive correlations between pregnancy/abortion frequency and maternal distress. Studies indicate that pregnancy loss exacerbates psychological burden during subsequent pregnancies, with emotional outcomes being moderated by maternal age, education level, and abortion frequency. Notably, the incidence of adverse emotional states shows a dose–response relationship with the number of prior abortions. Our findings demonstrate that birth weight significantly impacts infant outcomes, consistent with the results reported by Chang (21). Parents of low birth weight infants, particularly very low/extremely low birth weight infants, exhibit higher PTSD symptom prevalence compared to parents of normal birth weight preterm infants. Very low birth weight infants are more susceptible to prematurity-related complications, including sepsis, bronchopulmonary dysplasia, intraventricular hemorrhage, and necrotizing enterocolitis. Preterm birth and low birth weight may adversely affect neurodevelopmental and psychological outcomes, with failure to achieve catch-up growth potentially increasing risks for adult-onset conditions like diabetes and cardiovascular disease (22, 23). Furthermore, prolonged NICU stays resulting in mother–infant separation, combined with maternal inexperience in breastfeeding and post-discharge feeding anxieties (e.g., aspiration and cyanosis concerns), may exacerbate psychological distress and increase PTSD vulnerability.

According to Mishel’s uncertainty in illness theory (24), illness-related uncertainty impairs the ability of mothers of preterm infants to seek and process medical information, ultimately affecting maternal and infant health outcomes. In this study, the mother of a preterm infant scored 85 on the illness uncertainty scale, significantly higher than the threshold of 70, indicating a high level of uncertainty. Illness uncertainty is a risk factor for PTSD in these mothers, consistent with the findings of Gorsky. Among the four uncertainty dimensions, the highest average scores were in unpredictability and lack of information. Preterm infants with lower birth weights, more complex conditions, and greater complications heighten maternal uncertainty. Since all preterm infants in this study weighed <1,500 g, their mothers scored particularly high in unpredictability. The primary source of uncertainty stems from inadequate provider–patient communication: the elevated scores in the information deficiency dimension may reflect insufficient maternal health literacy.

This study found that mothers of preterm infants perceived stronger support from family [median score: 22 (IQR 18–25)] and friends [19 (16–24)] compared to other sources [e.g., relatives, colleagues; 19 (16–23)], suggesting that family and friends provide more substantial psychological support (25, 26), demonstrating that social support acts as a buffer between psychosocial stress and health outcomes, reducing the risk of PTSD. Given that very low birth weight infants typically require prolonged hospitalization and incur higher medical costs, robust social support becomes particularly crucial. Insufficient social support may elevate cortisol secretion and heighten biological stress sensitivity, whereas adequate support mitigates trauma perception (26, 27) and alleviates maternal psychological distress. Based on these findings, we propose the following strategies to reduce PTSD incidence among mothers of preterm infants: 1) family-level interventions: family members and friends—especially spouses—should provide consistent companionship, emotional support, and encouragement; 2) individual-level actions: mothers should recognize the value of social support, engage in community activities, and proactively seek help from their social networks to manage emotional distress; and 3) healthcare provider role: clinicians should screen for maternal distress early and facilitate peer support groups where mothers can share experiences, fostering confidence in their infant’s recovery and preventing PTSD through shared coping strategies.

Coping style is a critical determinant of outcomes following stressful events. Mothers of preterm infants experience chronic stress due to multiple factors: disease complexity, prolonged hospitalization, uncertain prognosis, restricted NICU access, high medical costs, and fear of unknown outcomes. Those who adopt negative coping strategies often develop anxiety, depression, and other adverse emotional states, which hinder their own recovery. Research indicates that positive coping strategies enhance psychological resilience and confidence when facing traumatic events, promoting both mental and physical health (28, 29). Therefore, healthcare providers should regularly assess mothers’ coping styles, offer disease-specific information and emotional support to families, and minimize ineffective coping behaviors.

Postpartum PTSD frequently presents with psychiatric comorbidities, particularly major depressive disorder and anxiety disorders. Existing literature demonstrates substantial comorbidity, with one study finding 90.4% of postpartum women with PTSD concurrently meeting the diagnostic criteria for major depression (30). Anxiety symptom elevation similarly demonstrates a high co-occurrence with PTSD in the perinatal period. A methodological limitation of our study was the absence of validated depression or anxiety assessment tools. This precluded both the quantification of comorbidity prevalence and statistical adjustment for these confounding factors. Future investigations should incorporate standardized measures (e.g., Edinburgh Postnatal Depression Scale, GAD-7) to better characterize these comorbidities and their interactions with PTSD.

PTSD in the postpartum period commonly co-occurs with other mental health conditions, especially depression and anxiety. Although our study focused on PTSD symptoms, comorbidity was high. For instance, one study reported that 90.4% of women with postpartum PTSD also met the criteria for major depression (31, 32). Similarly, elevated anxiety symptoms often accompany PTSD in new mothers. Our data collection did not include validated instruments for postpartum depression or anxiety. The absence of these measures limits our ability to quantify comorbidity rates or adjust for their influence. Future studies should administer standardized depression and anxiety scales to examine this overlap with PTSD.

Our scope was limited to PTSD as defined by symptoms persisting at least 1 month postpartum. We deliberately did not assess acute trauma reactions or grief-specific responses. Acute stress disorder (ASD) refers to PTSD-like symptoms occurring within the first month after trauma (33, 34). By requiring a 1-month interval, our protocol excluded cases of ASD. It is possible that some women experienced significant acute distress immediately postpartum that either resolved or progressed to full PTSD. Future studies might track early post-traumatic symptoms to predict later PTSD. Similarly, we did not specifically measure perinatal grief reactions (such as following miscarriage or stillbirth), which involved trauma and loss but were beyond our study’s focus.

Potential confounders and effect modifiers were not all accounted for in our analysis. In particular, social support and coping style can significantly influence PTSD risk. Adequate social support during and after childbirth generally protects against PTSD, whereas low support elevates risk (35, 36). Similarly, adaptive coping strategies mitigate trauma impact, while maladaptive coping (e.g., avoidance) can exacerbate symptoms (37). We did not measure these psychosocial factors, so differences in support or coping between groups could confound our observed associations.

Postpartum depression and anxiety are highly prevalent among mothers with PTSD. Due to the absence of depression and anxiety screening tools in this study, we acknowledge this as a limitation and recommend inclusion in future research to fully evaluate comorbidity effects.

Our study evaluated PTSD symptoms at a follow-up point beyond 1 month postpartum and did not focus on acute stress reaction (within days) or acute PTSD (within 1 month). Furthermore, perinatal grief—typically linked to fetal or neonatal loss—was not assessed, as our cohort involved surviving infants. However, emotional responses related to threatened loss or severe neonatal complications may mimic grief processes, suggesting a need for future investigation (38).

Several potential confounders and effect modifiers—such as maternal age, socioeconomic status, obstetric history, and social support—may influence PTSD risk. While our regression model controlled for many of these variables, unmeasured confounding may remain. We also note that social support and coping styles may interact with stress exposure to modify PTSD risk.

## Conclusion

Through rigorous methodology, we developed and validated a clinically useful nomogram for predicting PTSD risk in mothers of ELBWIs. This practical scoring tool enables clinicians to identify high-risk mothers efficiently and to optimize their clinical management. The model provides valuable early warning capabilities and enhances screening for high-risk cases, representing an important advancement in maternal mental healthcare.

### Research innovations

1. Utilizing a prospective design, this study provides a comprehensive systematic evaluation of risk factors for PTSD in mothers of VLBWIs, synthesizing domestic and international evidence to facilitate early identification of high-risk populations.
2. This represents the first application of a nomogram for visual risk prediction of PTSD in mothers of VLBWIs, offering clinicians an intuitive, efficient tool for individualized risk assessment.

### Study limitations

1. The study’s relatively small sample size and single-region hospital recruitment may limit the generalizability of the findings.
2. Reliance on self-reported questionnaire data introduces potential reporting biases, including possible overreporting of symptoms.
3. The nomogram requires external validation in diverse populations to confirm its clinical utility.

## Data Availability

The original contributions presented in the study are included in the article/supplementary material. Further inquiries can be directed to the corresponding authors.
